# Animal Allergens: Common Protein Characteristics Featuring Their Allergenicity

**DOI:** 10.3389/fimmu.2015.00040

**Published:** 2015-02-05

**Authors:** Annette Kuehn, Christiane Hilger

**Affiliations:** ^1^Department of Infection & Immunity, Luxembourg Institute of Health, Esch-sur-Alzette, Luxembourg

**Keywords:** animal allergen exposure, allergenicity, allergy, biological function, cross-reactivity, immune response, isoallergens, immunotherapy

Allergy to proteins and glycoproteins from animal sources is an important public health problem affecting both children and adults (1–3). Exposure to animal allergens is a key factor for the development of allergy, particularly for the development of allergic airway diseases such as asthma and rhinitis (4, 5). Among the many particles and molecules of animal origin present in our environment, some have the property to induce allergic sensitization and IgE-mediated reactions. Major animal allergens share common protein characteristics as they belong to specific protein families: a majority of inhalant mammalian allergens belong to the lipocalin and serum albumin families. Many mite allergens are proteases, food allergens are often EF-hand proteins, tropomyosins, or caseins (6–8). These protein family clusters observed in allergens suggest that common structural, biochemical, or functional features in addition to external adjuvant factors contribute to their allergenicity. The understanding of the nature their allergenicity is of high importance as it contributes to the development of future diagnostic tools and therapeutic strategies (9–12).

The present compilation of review articles illustrates that animal allergens are ubiquitous. The human immune system is exposed to these proteins not only via the respiratory tract such as for pet and house dust mite allergens via the intestine such as for food and helminth allergens but also via the skin such as for insect allergens.

The extensive review of Zahradnik and Raulf focuses on animal allergens from furry animals in indoor (cat, dog) and outdoor environment (horse, cattle). Allergies to these animal proteins are a public problem as exposure is not limited to the original source but as these molecules become airborne and are carried on clothing to public buildings, the general population is exposed to these allergens. Allergen detection in the private or occupational context is therefore an important first step to develop avoidance strategies for patients at risk.

Díaz-Perales et al. present an overview of uncommon allergenic sources such as exotic pets, which are on the rise as elicitors of animal allergies. These sources are to be considered as relevant because on one hand, their numbers are progressively increasing in households and on the other hand, cross-reactivity to common companion animals via molecules of conserved protein families might occur.

Hentges and colleagues approach the topic of animal allergens from another point of view by focusing on common features of the human immune response to three allergen families, namely, secretoglobins, lipocalins, and serum albumins. Mammalian allergens initiate a broad variety of immunological processes in sensitized patients. The understanding of these mechanisms is crucial for defining strategies of future immunotherapies.

Dumez et al. refer to the most frequent animal elicitors of allergy, which are house dust mites. Among the spectrum of identified mite allergens, they focus on the allergens’ biological function in the context of activation pathways initiating the underlying immune response during sensitization. The answer to the crucial question “why is an allergen an allergen?” seems to be well advanced in this context. This highlights the need for such functional analysis for further animal allergens.

Fitzsimmons, Falcone, and Dunne report on allergens from helminths, which provoke strongly skewed Th2 responses as a normal physiological response upon parasitic infection. This review touches another important aspect: a reduced allergy prevalence has been associated with helminth endemic areas. But on the other hand, many environmental animal allergens have homologs in metazoan parasites, which may lead to cross-reactivity and increase of allergy prevalence. As a perspective, further elucidation of the immunological processes during parasite infection may improve insights for the development of therapeutic strategies in allergy.

Kuehn and colleagues target another important source of animal allergens from food origin, namely fish allergens, which enter the human body by ingestion, inhalation, or skin contact. Molecular and immunological characteristic of the major fish allergens, parvalbumins, as well as the recently described enolases and aldolases, are in the focus while highlighting the variable allergenicity of closely related isoallergens and providing a basis for further studies of the mechanism of sensitization and allergy.

Spillner, Blank, and Jakob detail the recent rapid advances in the characterization of hymenoptera venom allergens. The availability of a substantial number of isolated and recombinant allergens allowed to define individual patient IgE-profiles, to predict cross-reactivity and clinical cross-sensitization. The use of an increasing molecular allergen panel will ultimately allow to monitor and assess therapeutic outcome.

Salazar and Ghaemmaghami report on the role of dendritic and epithelial cells in the process of allergic sensitization. They focus on how different receptors such as C-type lectin, toll-like and protease activated receptors, and the associated signaling pathways contribute to the sensitization process. The elucidation of the molecular mechanisms of dendritic and epithelial cell cross-talk in the process of sensitization will hopefully lead to the development of new therapeutic applications.

Van Hage and Pauli finally summarize the recent advances in immunotherapy for mammalian allergy. Most studies focused on Fel d 1, the major cat allergen, as it is a well characterized allergen in terms of B- and T-cell epitopes. Different molecular design strategies, the use of the molecules in mouse models as well as in clinical trials and therapeutic outcomes are presented. The field is evolving rapidly and further studies are needed to assess safety and efficacy of the new molecules.

We are thankful to all colleagues who contributed to this Research Topic, without their highly valuable expertise in different fields of research on animal allergen research it would not have been possible to realize this issue.

## Conflict of Interest Statement

The authors declare that the research was conducted in the absence of any commercial or financial relationships that could be construed as a potential conflict of interest.
